# Supplementary material for: A tri-linear quantum dot architecture for semiconductor spin qubits
Source: Sci Rep. 2026 Apr 6;16:16526. doi: 10.1038/s41598-026-42575-z (PMC13216268; doi:10.1038/s41598-026-42575-z)
Supplement: Supplementary file 1 — Supplementary Material 1 [file 41598_2026_42575_MOESM1_ESM.pdf]

Supplementary information
A tri-linear quantum dot architecture for semiconductor spin qubits

R. Li*[1], V. Levajac[1,3], C. Godfrin[1], S. Kubicek[1], G. Simion[1], B. Raes[1], S. Beyne[1], I. Fattal[1,2], A. Loenders[1,2], W. De Roeck[3], M. Mongillo[1], D. Wan[1], K. De Greve[1, 2]

[1] IMEC, Leuven, Belgium
[2] Department of Electrical Engineering, KU Leuven, Leuven, Belgium
[3] Department of Physics, KU Leuven, Leuven, Belgium

Section 1, Sample implementation of a fully functional 1024 qubit chip based on the tri-linear architecture

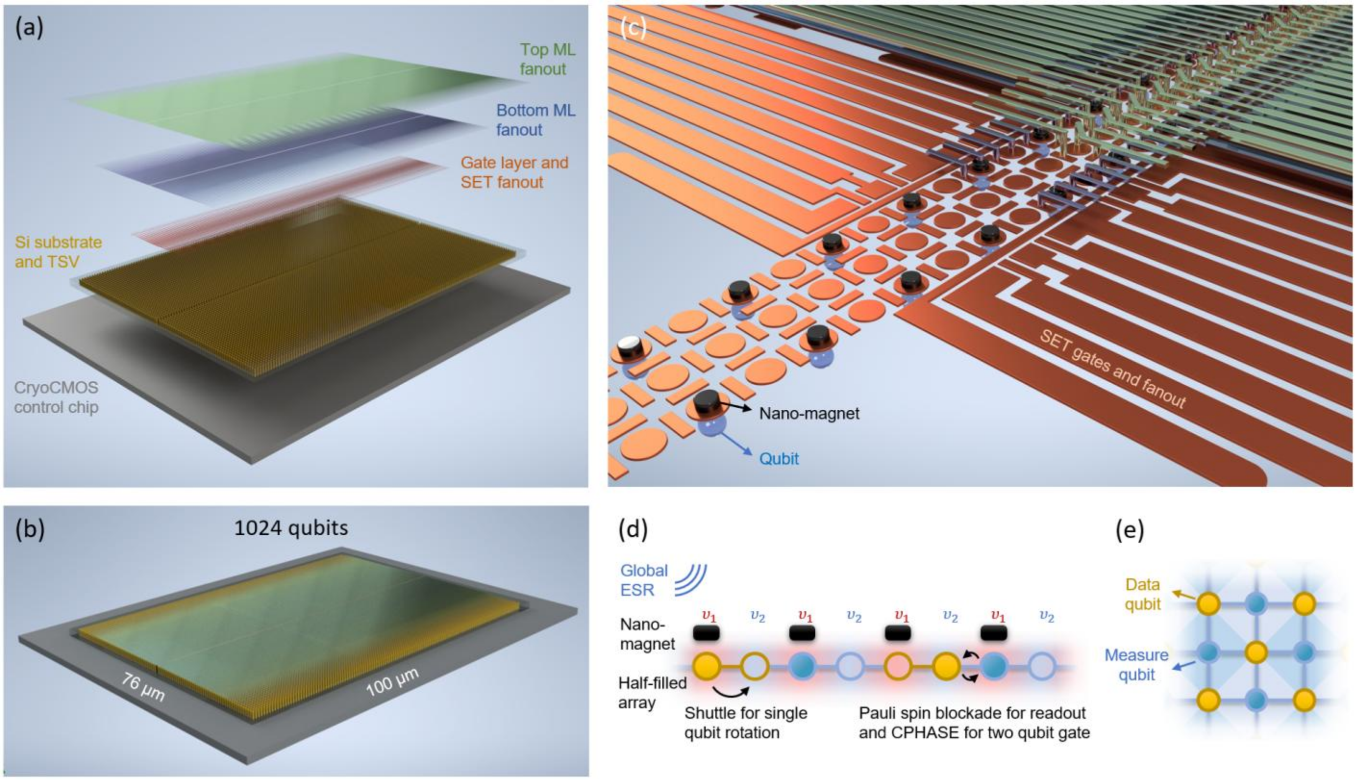

Supplementary Fig. 1 An example layout of a fully functional 1024-qubit chip. (a) The layered breakdown of the qubit chip, including, from bottom to top, (i) the cryoCMOS control chip, (ii) the qubit-hosting silicon substrate and TSVs which connect the control wiring from the cryoCMOS to each quantum dot and SET gate, (iii) the quantum dot and SET gates and fanout connection to TSVs, (iv) the bottom metal layer and fanout, and (v) the top metal layer and fanout. **(b)** A 3D schematic of the qubit chip. For 1024 qubits, the qubit array and gate fanout size is ~ 76 µm × 100 µm. **(c)** A zoomed-in image of the tri-linear array. SETs are attached to both sides of the tri-linear array for charge sensing. On every other quantum dot in the qubit array, a nanomagnet is placed on top of the plunger gate, which is also used as electrical connection to that plunger gate. **(d)** The operation scheme of the qubit chip. **(e)** The corresponding 2D grid where the blue and yellow colored qubits can be used as data and measure qubits for surface code implementations.

Here we show an example implementation of a fully functional qubit chip containing 1024 qubits. The 3D schematics are shown in Fig. S1 a-c. Most of the chip area is used for wiring fanout. The length of the vertical wiring fanout to the tri-linear array is set by the size of the TSVs, which have a pitch of 0.8 µm as used in the schematic plots.

Qubit operations are based on a half-filled array[1] with periodic local magnetic fields as shown in Fig. S1d. Applying an out-of-plane static magnetic field defines the spin quantization axis. A nanomagnet is placed on top of every other plunger gate in the 1D quantum dot (QD) array. This arrangement of nanomagnets generates a periodic magnetic field oscillation along the array between resonance frequencies ν_1 and ν_2, giving rise to two different qubit frequencies depending on if a QD is under a nanomagnet or not. We propose a realization where QD arrays are half-filled with qubits, with QDs under nanomagnets (ν_1) having qubits and QDs without nanomagnets (ν_2) kept empty. When a ν_2-resonant global ESR $\nu_2$is applied, a qubit can be shuttled to its neighbouring nanomagnet-less QD where it will be resonant with the ESR and exhibit a single qubit gate rotation. In this way, qubits can be selectively targeted to either idle in QDs or shuttle to empty QDs to be operated on, thereby allowing addressable single qubit gate operations with a global ESR. The frequency difference between ν_1 and ν_2 also allows two-qubit gate operations via CPHASE and Pauli spin blockade-based readout. Along a 1D array, a qubit could alternatively serve as data and measurement qubit. Extrapolating to 2D, as shown in Fig. S1e, would allow for surface code operations[2].

It is worth noting that shutting in the middle quantum dot array could cause additional Z rotations. Nonetheless, this rotation can be calibrated and compensated (e.g. by virtual Z gates).

Section 2, Wiring interconnect and multiplexing scheme with cryoCMOS

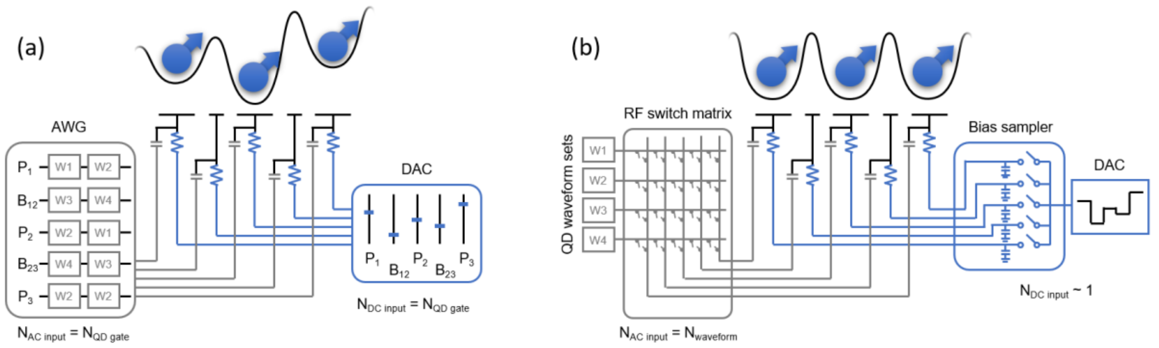

Supplementary Fig. 2 Qubit wiring and control schemes. (a) The conventional wiring scheme, where separated DAC and AWG channels are needed for each quantum dot gate. **(b)** The multiplexing scheme with a cryoCMOS chip, where a fixed number of AC and DC inputs are needed to control a large qubit array.

To optimize qubit operation fidelities, typically each gate needs dedicated AC and DC controls, as shown in Fig. S2a. To achieve a scalable wiring and interconnect scheme, which should scale sub-linearly with the number of qubits[3], a cryoCMOS circuits chip is proposed, as shown in Fig. S2b. The cryoCMOS chip contains a bias sampler and an RF switch matrix. For each dot gate, the bias sampler uses a switch to couple an external DAC for voltage biasing, and charge storing capacitors store the voltage after opening the switch and decoupling from the DAC. This scheme is known as the floating gate scheme[4]. With low charge leakage rate on the capacitor and the quantum dot gate (more than one hour holding time)[5], a single DC input could support hundreds of quantum dot gates by sequentially changing the DAC voltage to the corresponding quantum dot gate voltage once per second, for example, and then refreshing the corresponding floating gate capacitor.

For AC input, we assume that once the static biasing point is corrected by the floating gate DC bias, the same AC pulse can be used across all quantum dot gates. For typical qubit operations, only a limited set of waveforms are required, including qubit shuttling in the middle dot array (e.g. 4 waveforms are needed for conveyor-mode shuttling[6]), single qubit hopping in the outer dot arrays, two qubit gates,

readouts, and each compensation gate pulses. These waveforms can be periodically generated with external AC inputs and selectively distributed to different quantum dot gates (and simultaneously for parallel qubit operation) through the RF switch matrix. In this case, even with increased qubit numbers, the number of AC inputs is fixed (as the total required waveforms is not related to the qubit array size).

The above discussion assumes that the quantum dots have good uniformity for shared AC control (or good capacitance matrix uniformity to be more specific[7]). Meanwhile, if there are few outlier dots, more input channels can be included on the RF switch matrix to allows for dedicated AC control input for the outlier dots. If the uniformity of the dots is not sufficiently good, time-domain multiplexing, or row by row operation has to be used[8].

Section 3, Sample loop connection scheme for the tri-linear array

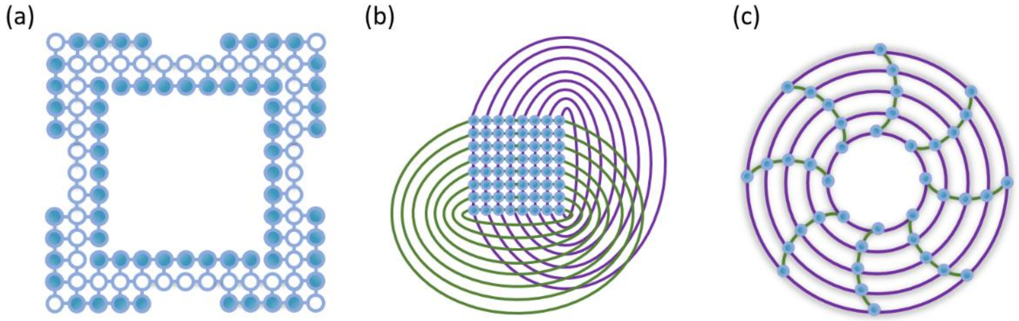

Supplementary Fig. 3 Loop connection scheme for the tri-linear array. (a) The schematic layout of a tri-linear array that is equivalent to an 8×8 2D array. The head and tail of the tri-linear array are connected together, forming a loop. **(b)** The equivalent connection scheme of (a). The green lines show the connection within the same row by a shuttling length of $2\sqrt{N}$, same as the green line in main text Fig. 4c. The purple lines show the connection of column heads and tails through the loop of the connection of the tri-linear array. **(c)** The donut equivalent connection scheme of (a) and (b), where the standard 2D array edge is eliminated. Note that qubits and connections at the backside of the donut are not shown.

Fig S3 shows the connection scheme of the tri-linear array to form a donut to eliminate all edges on a square 2D array. Here we use an 8×8 2D array for illustration purposes. Larger arrays could allow for even easier layout as well as more space inside the loop for fanout. Moreover, by curving the tri-linear array or meandering the array, the chip areas can be more effectively utilized for large systems, as the straight 1D layout can end up very long.

Supplementary references

1. Simion, G. *et al.* A Scalable One Dimensional Silicon Qubit Array with Nanomagnets. in *2020 IEEE International Electron Devices Meeting (IEDM)* 30.2.1-30.2.4 (2020). doi:10.1109/IEDM13553.2020.9372067.
2. Fowler, A. G. Surface codes: Towards practical large-scale quantum computation. *Phys. Rev. A* **86**, (2012).
3. Franke, D. P., Clarke, J. S., Vandersypen, L. M. K. & Veldhorst, M. Rent's rule and extensibility in quantum computing. *Microprocess. Microsyst.* **67**, 1–7 (2019).
4. Vandersypen, L. M. K. *et al.* Interfacing spin qubits in quantum dots and donors—hot, dense, and coherent. *Npj Quantum Inf.* **3**, 34 (2017).
5. Li, R. *et al*. Stable floating-gate control of Si qubit devices with cryoCMOS circuits. Silicon Quantum Electronics Workshop (SiQEW) in Kyoto International Conference Center, 2023.

6. De Smet, M. *et al.* High-fidelity single-spin shuttling in silicon. Preprint at https://doi.org/10.48550/ARXIV.2406.07267 (2024).
7. Hanson, R., Kouwenhoven, L. P., Petta, J. R., Tarucha, S. & Vandersypen, L. M. K. Spins in few-electron quantum dots. *Rev. Mod. Phys.* **79**, 1217–1265 (2007).
8. Li, R. *et al.* A crossbar network for silicon quantum dot qubits. *Sci. Adv.* **4**, eaar3960 (2018).
